# Diagnostic stewardship for blood cultures in the emergency department: A multicenter validation and prospective evaluation of a machine learning prediction tool

**DOI:** 10.1016/j.ebiom.2022.104176

**Published:** 2022-07-16

**Authors:** Michiel Schinkel, Anneroos W. Boerman, Frank C. Bennis, Tanca C. Minderhoud, Mei Lie, Hessel Peters-Sengers, Frits Holleman, Rogier P. Schade, Robert de Jonge, W. Joost Wiersinga, Prabath W.B. Nanayakkara

**Affiliations:** a Section General Internal Medicine, Department of Internal Medicine, Amsterdam Public Health Research Institute, Amsterdam UMC, location VU University Medical Center, De Boelelaan 1118, 1081 HZ Amsterdam, the Netherlands; b Center for Experimental and Molecular Medicine (CEMM), Amsterdam UMC, location Academic Medical Center, Meibergdreef 9, 1105 AZ Amsterdam, the Netherlands; c Department of Clinical Chemistry, Amsterdam UMC, Vrije Universiteit Amsterdam, AGEM Research Institute, De Boelelaan 1118, 1081 HZ Amsterdam, the Netherlands; d Department of Computer Science, Quantitative Data Analytics Group, Department of Computer Science, Faculty of Science, VU University, De Boelelaan 1105, 1081HV Amsterdam, the Netherlands; e Department of EVA Service Center, Amsterdam UMC, location VU University Medical Center, De Boelelaan 1118, 1081 HZ Amsterdam, the Netherlands; f Department of EVA Service Center, Amsterdam UMC, location Academic Medical Center, Meibergdreef 9, 1105 AZ Amsterdam, the Netherlands; g Section General and Acute Internal Medicine, Department of Internal Medicine, Amsterdam UMC, location Academic Medical Center, Meibergdreef 9, 1105 AZ Amsterdam, the Netherlands; h Department of Medical Microbiology and Infection Prevention, Amsterdam UMC, location Academic Medical Center, Meibergdreef 9, 1105 AZ Amsterdam, the Netherlands; i Section Infectious Diseases, Department of Internal Medicine, Amsterdam UMC, location Academic Medical Center, Meibergdreef 9, 1105 AZ Amsterdam, the Netherlands

**Keywords:** Blood cultures, Machine learning, Emergency department, Validation, Decision-curve analysis

## Abstract

**Background:**

Overuse of blood cultures (BCs) in emergency departments (EDs) leads to low yields and high numbers of contaminated cultures, accompanied by increased diagnostics, antibiotic usage, prolonged hospitalization, and mortality. We aimed to simplify and validate a recently developed machine learning model to help safely withhold BC testing in low-risk patients.

**Methods:**

We extracted data from the electronic health records (EHR) for 44.123 unique ED visits with BC sampling in the Amsterdam UMC (locations VUMC and AMC; the Netherlands), Zaans Medical Center (ZMC; the Netherlands), and Beth Israel Deaconess Medical Center (BIDMC; United States) in periods between 2011 and 2021. We trained a machine learning model on the VUMC data to predict blood culture outcomes and validated it in the AMC, ZMC, and BIDMC with subsequent real-time prospective evaluation in the VUMC.

**Findings:**

The model had an Area Under the Receiver Operating Characteristics curve (AUROC) of 0.81 (95%-CI = 0.78–0.83) in the VUMC test set. The most important predictors were temperature, creatinine, and C-reactive protein. The AUROCs in the validation cohorts were 0.80 (AMC; 0.78–0.82), 0.76 (ZMC; 0.74–0.78), and 0.75 (BIDMC; 0.74–0.76). During real-time prospective evaluation in the EHR of the VUMC, it reached an AUROC of 0.76 (0.71–0.81) among 590 patients with BC draws in the ED. The prospective evaluation showed that the model can be used to safely withhold blood culture analyses in at least 30% of patients in the ED.

**Interpretation:**

We developed a machine learning model to predict blood culture outcomes in the ED, which retained its performance during external validation and real-time prospective evaluation. Our model can identify patients at low risk of having a positive blood culture. Using the model in practice can significantly reduce the number of blood culture analyses and thus avoid the hidden costs of false-positive culture results.

**Funding:**

This research project was funded by the Amsterdam Public Health – Quality of Care program and the Dutch “Doen of Laten” project (project number: 839205002).

**Research in context**

**Evidence before this study**

We performed a Pubmed title/abstract search on January 18^th^, 2022, using the terms “Bacteremia” OR “Bacteraemia” OR “Bloodstream Infection” AND “Machine Learning” OR “Prediction” AND “Emergency Department.” The search yielded 62 papers, and we found additional articles through the references. The literature shows that various (machine learning) prediction tools for blood cultures outcomes in the emergency department (ED) have been developed. Most studies only describe the model development, while the few externally validated models are tested in at most one other center. Only the Shapiro Decision Rule seems to have made it into clinical practice.

**Added value of this study**

We have created a robust tool for predicting blood culture outcomes in the ED. The tool was validated in multiple geographical locations and various types of hospitals during the development phase and subsequently prospectively evaluated in real-time. We demonstrated a net benefit of using this tool during the real-time evaluation with a decision-curve analysis.

**Implications of all the available evidence**

The literature suggests that it is possible to predict the outcome of a blood culture that is drawn in the ED. This information can be used to substantially and safely reduce unnecessary blood culture analyses and avoid the hidden costs of false-positive culture results. We now present a robust tool that can be easily implemented in various settings, and which is already implemented in the VUMC electronic health record environment. The tool is ready to be tested in a clinical trial to formally study its impact on clinical practice.

Alt-text: Unlabelled box

## Introduction

Blood cultures are indispensable for diagnosing bloodstream infections (BSIs), ranking among the top seven causes of death in most European and North American countries.^1^ An estimated 536.000–628.000 episodes of BSI occur annually in the United States alone, with 79.000–94.000 associated deaths.^1^ Physicians tend to order blood cultures frequently due to the fear of missing such a severe but treatable condition.^2^^,^^3^ In emergency departments (EDs), blood cultures are collected in many patients with suspected infections, even when the primary condition is one with a low probability of being accompanied by bacteremia, such as pneumonia or cellulitis.^2^^,^^3^ Consequently, the yield of blood cultures in the ED is low.^3^ The percentage of true-positive blood cultures, disregarding contamination, ranges from 1.4% to 12.2% in ED populations worldwide.^4^, ^5^, ^6^, ^7^, ^8^, ^9^, ^10^ Due to these low yields, blood culture outcomes affect treatment decisions in only 0.18–2.8% of patients presenting to the ED with suspected infection.^4^^,^^5^

The primary goal of blood culture testing should be to maximize the identification of true BSIs. However, testing all patients with suspected infections has unwanted consequences.^11^ The abundant use of blood cultures leads to unnecessarily high numbers of contaminated cultures. A substantial 40–55% of positive cultures can be contaminated.^5^^,^^6^^,^^8^^,^^9^^,^^12^ Three decades of research on this topic has consistently shown that contamination is associated with additional resource use (laboratory and microbiological testing), increased use of antibiotics, prolonged hospital stay, and even increased in-hospital mortality.^9^^,^^12^, ^13^, ^14^

Diagnostic stewardship interventions that provide a swift and personalized blood culture testing approach are urgently needed to reduce the overuse of blood cultures and the serious secondary effects of contamination.^15^ We recently demonstrated the feasibility of using electronic health record (EHR) data in a machine learning model to detect patients at low risk of a positive blood culture, in whom blood culture analyses could safely be avoided.^10^ However, this model did not lend itself well to external validation and clinical implementation due to the many features included. The current study aimed to create a simplified machine learning-based blood culture prediction tool that only uses patient characteristics, vital sign measurements, and routine laboratory results to facilitate clinical use and implementation in other hospitals. To examine the performance of this model in different care settings, we carried out a multicenter external validation in academic and teaching hospitals in various geographical locations. We also evaluated the predictions prospectively in the EHR environment and performed a decision curve analysis to establish the tool's potential net benefit to safely reduce unnecessary blood cultures.

## Methods

### Study design, population, and data sources

We performed a retrospective multicenter study with EHR data collected from four hospitals to develop and validate a logistic regression model and a gradient-boosting decision tree model (XGBoost) for blood cultures results in the ED. The better performing XGBoost model was subsequently subjected to a prospective single-center real-time evaluation. This study adheres to the “transparent reporting of a multivariable prediction model for individual prognosis or diagnosis (TRIPOD)”.^16^

Patients were included if they were 18 years or older and underwent blood culture sampling during their ED stay. Data for developing the blood culture prediction models were extracted from the Amsterdam UMC - location VU Medical Center (VUMC) EHR system between 2016 and 2021. External validation data were extracted from the EHR systems of Amsterdam UMC – location Academic Medical Center (AMC; between 2020 and 2021) and the Zaans Medical Center (ZMC; between 2016 and 2021). We further validated the models on data from the Beth Israel Deaconess Medical Center (BIDMC; Boston, Massachusetts, United States) between 2011 and 2019, available to researchers in the online MIMIC-IV-ED database.^17^ The VUMC and AMC are academic hospitals, while the ZMC and BIDMC are teaching hospitals.

For prospective real-time evaluation, the XGBoost model was further integrated into the VUMC EHR environment from EPIC (EPIC Systems Corporation, Verona, Wisconsin, United States). The model predicted blood culture results for all adults who underwent blood culture sampling in the ED. The model started predicting the probability of a positive blood culture as soon as sufficient variables were documented in the EHR (see e-Methods section on patient selection for further explanation) and updated the prediction whenever additional results came in. For the prospective evaluation in this study, we analyzed all results between October 19^th^, 2021, and January 25^th^, 2022. Before the patients were either admitted or discharged from the ED, the final prediction was used to evaluate the model's performance. Notably, the predictions were registered in the EHR but not visible to the physicians.

### Variable selection and data preprocessing

The candidate variable selection, guided by our aim to simplify the machine learning model we created earlier, was based on the VUMC cohort.^10^ We selected age, sex, vital sign measurements, and laboratory results. These variable groups were the primary predictors in the initial model and are readily available in most hospitals.^10^ Based on the timestamps in the EHR, we selected only the vital signs and laboratory results that were registered in the system before the end of the ED visit. We selected laboratory tests measured in more than 50% of the patients as predictor variables. Other selection decisions were made to facilitate easy integration in different hospital systems, as discussed in the e-Methods. The most important of these selection decisions was that we only selected patient visits in which at least 20% of the vital sign data and 20% of the laboratory results were available for the prediction. Missing data was further handled using median imputation in combination with indicator variables (which indicate whether a value was measured (1) or not (0) on a patient-level), which is especially effective with data missing not at random, as is the case in our data.^18^ The AMC, ZMC, and BIDMC datasets were processed similarly, and the complete preprocessing pipeline is discussed in more detail in the e-Methods of the supplementary appendix, where we also reference all the packages, modules, and libraries that were used. We cleaned the data using the R statistical software version 3.6.1 (R Foundation for Statistical Computing, Vienna, Austria).

### Outcome

The outcome of interest was a BSI, defined as the growth of a clinically relevant pathogen in at least one blood culture bottle collected during the ED visit. Among the cultured microorganisms, we defined contaminants based on previous literature and classified those as negative cultures.^2^^,^^6^^,^^10^^,^^19^^,^^20^ e-Table 5 lists all organisms that were classified as contaminants. We also experimented with a contamination classification based on the number of bottles that grew a particular pathogen, highlighted in the e-Methods.

### Statistics

#### Model development and validation

For the model development and validation, we used Python version 3.8.1. The VUMC cohort was randomly split into a training (80%) and test set (20%), stratified by the blood culture outcomes. Subsequently, the training data were scaled to unit variance and imputed when missing. The same standardization factor and medians of the training data were used to scale and impute the test and validation data. As with our earlier approach, we trained a logistic regression model and a gradient-boosting decision tree model, implemented through Python's XGBoost (eXtreme Gradient Boosting; XGB) library.^10^ The optimal hyperparameters for both models were found through a fivefold cross-validated grid search (see e-Table 2 for further details).

We validated the models derived from the VUMC training set in the VUMC test set, AMC, ZMC, and BIDMC datasets. Therefore, according to the TRIPOD criteria, our study can be classified as both a type 2a and type 3 prediction model study.^16^ The discriminatory performances were assessed using the Area Under the curve of the Receiver Operating Characteristics (AUROC) and the Area Under the Precision-Recall Curve (AUPRC). The AUPRC is more robust to class imbalances, as we see with the low incidence of positive blood culture outcomes.^21^ The model calibration was assessed visually using calibration plots. Feature contributions for the logistic regression were presented using the coefficients, and those of the XGBoost model were reported using Shapley values, which correspond to the local contributions of the features for each prediction.^22^

On top of evaluating the model's performance, we analyzed the potential clinical net benefit through a decision curve analysis of the prospective real-time evaluation, as recommended by editorials in leading medical journals.^23^ The net benefit decision curve analysis takes into account the relative impact of false negatives (i.e., missing a BSI) and false positives (i.e., more contaminated cultures, with associated side-effects) for a range of threshold probabilities.^23^^,^^24^ A detailed description of the net benefit calculations can be found in the e-Methods.

### Ethics

The Amsterdam University Medical Centers’ (UMC) local medical ethics review committee waived the review of the retrospective and prospective part of this study (IRB number: IRB00002991; case: 2020.486), as the medical research involving Human Subjects Act did not apply. De-identified data extracts were used for this study, adhering to the local privacy officer's protocol. Therefore, no informed consent needed to be obtained for the use of the data. Participant data underlying the results of this study can be shared. The data can be requested following publication of this work. The data can be shared with researchers who provide a methodologically sound proposal, which is allowed under our local privacy regulations. Proposals should be directed to the corresponding author and requestors will need to sign a data access agreement. Part of the data is available to all researchers through the MIMIC-IV-ED database (https://physionet.org/content/mimic-iv-ed/1.0)

### Role of funding source

The funding sources (Amsterdam Public Health – Quality of Care program and the “Doen of Laten” project (project number: 839205002)) had no involvement in any part of the research project and did not have any influence on the decision to submit the work for publication.

## Results

### Cohort description

This multicenter development and validation study used retrospective EHR data from four hospitals (VUMC, AMC, ZMC, and BIDMC) where patients with all categories of diseases and severity presented at the ED. After selecting only adult patients who underwent blood culture sampling during their ED stay and who had over 20% of the vital signs and 20% of the laboratory variables measured, the VUMC cohort consisted of 8.027 unique visits, of whom 6.421 were randomly allocated to the training set and 1.606 to the test set. The validation cohort sizes were 2.429 (AMC), 5.961 (ZMC), and 27.706 (BIDMC). The percentage of true-positive blood cultures ranged from 5.4% (BIDMC) to 12.3% (ZMC). The percentage of contaminated cultures, which we later classified as negative, ranged from 4.9% (BIDMC) to 10.6% (AMC). Detailed information about the predictor variables and outcomes in the different cohorts is presented in Table 1. The number of ED visits included following each step of the selection procedure is presented in e-Figure 1 and frequently found microorganisms in the different cohorts are presented in e-Figure 7.

**Table 1:** Cohort descriptions of predictor variables and outcomes in the datasets used to develop and validate the XGBoost model to predict blood culture outcomes in the emergency department.

Variable	VUMC training (*n*=6.421)	VUMC test (*n*=1.606)	AMC (*n*=2.429)	ZMC (*n*=5.961)	BIDMC (*n*=27.706)
Age, median, *y* (IQR)	66 (52–76)	66 (53–76)	62 (48–73)	71 (58–81)	61 (49–73)
Sex, Female, *n* (%)	3666 (43.2%)	896 (44.2%)	1134 (46.7%)	2770 (46.5%)	14,075 (50.8%)
**Vital signs, median (IQR)**					
Temperature, Celsius	37.7 (36.9–38.5)	37.8 (36.9–38.5)	37.0 (36.3–37.7)	37.4 (36.6–38.3)	36.8 (36.6–37.1)
Heart rate, /min	94 (81–106)	93 (81–105)	90 (78–102)	95 (83–109)	85 (74–96)
Systolic blood pressure, mmHg	124 (110–140)	123 (110–140)	128 (113–144)	129 (114–145)	125 (112–139)
Diastolic blood pressure, mmHg	74 (66–83)	74 (65–83)	76 (67–85)	78 (68–87)	70 (62–78)
Respiratory rate, /min	20 (16–25)	20 (16–25)	20 (16–24)	20 (16–25)	18 (16–19)
Saturation, %	96 (95–98)	96 (94–98)	97 (95–98)	96 (93–98)	98 (96–99)
**Laboratory results, median (IQR)**					
C-Reactive Protein	63 (21–141)	58 (19–142)	46 (12–115)	69 (28–160)	48 (11–113)
Creatinine	85 (66–119)	84 (65–116)	88 (69–129)	85 (67–114)	88 (62–133)
Leukocytes	10.4 (7.0–14.5)	10.3 (6.9–14.5)	9.1 (6.2–13.1)	10.6 (7.3–14.75)	9.3 (6.6–12.9)
**Outcome**					
Positive blood cultures, %	11.5	11.5	11.2	12.3	5.4
Contaminated cultures, %	6.3	6.3	10.6	5.2	4.9

IQR = Interquartile Range; VUMC = VU Medical Center; AMC = Academic Medical Center; ZMC = Zaans Medical Center; BIDMC = Beth Israel Deaconess Medical Center.

### Training performances during the model development

**Figure 1:**
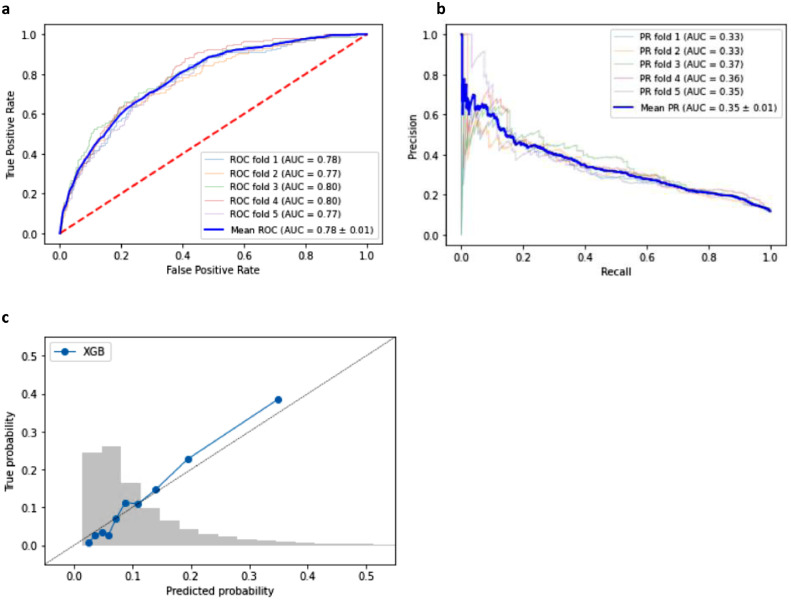
Discriminatory performance and calibration of the XGBoost model for predicting the outcome of blood cultures in the emergency department in the VUMC training set: a. the area under the receiver operating characteristics curve (AUROC). b. the area under the precision-recall curve (AUPRC). c. the calibration plot of predicted probabilities compared with actual probabilities. In grey, we further see a histogram of the distribution of the predictions in the training set in this figure.

Based on the AUROC and AUPRC, the XGBoost model consistently outperformed the logistic regression model. Therefore, we only present the XGBoost model performances here. A detailed description of the logistic regression model performance can be found in the supplementary appendix. The XGBoost model reached an average AUROC of 0.78 (standard deviation (SD) = 0.01) and an AUPRC of 0.34 (SD = 0.01) during the training phase, visualized in Figure 1a and 1a. The calibration plot, presented in Figure 1c, shows that the model is well-calibrated. Notably, the calibration plot comprises ten bins of equal population size. High probabilities were rare, as shown in the grey histogram of the prediction distributions in Figure 1c.

### Features and feature importances

Based on the VUMC development cohort, we selected age, sex, six vital sign measurements, and eighteen laboratory tests as predictor variables in the model. With an additional 23 indicator variables, the model included 49 features. Details on the percentage of imputed values per feature are presented in e-Table 3. Summary statistics of the features, stratified by blood culture outcome, are presented in e-Table 4.

**Figure 2:**
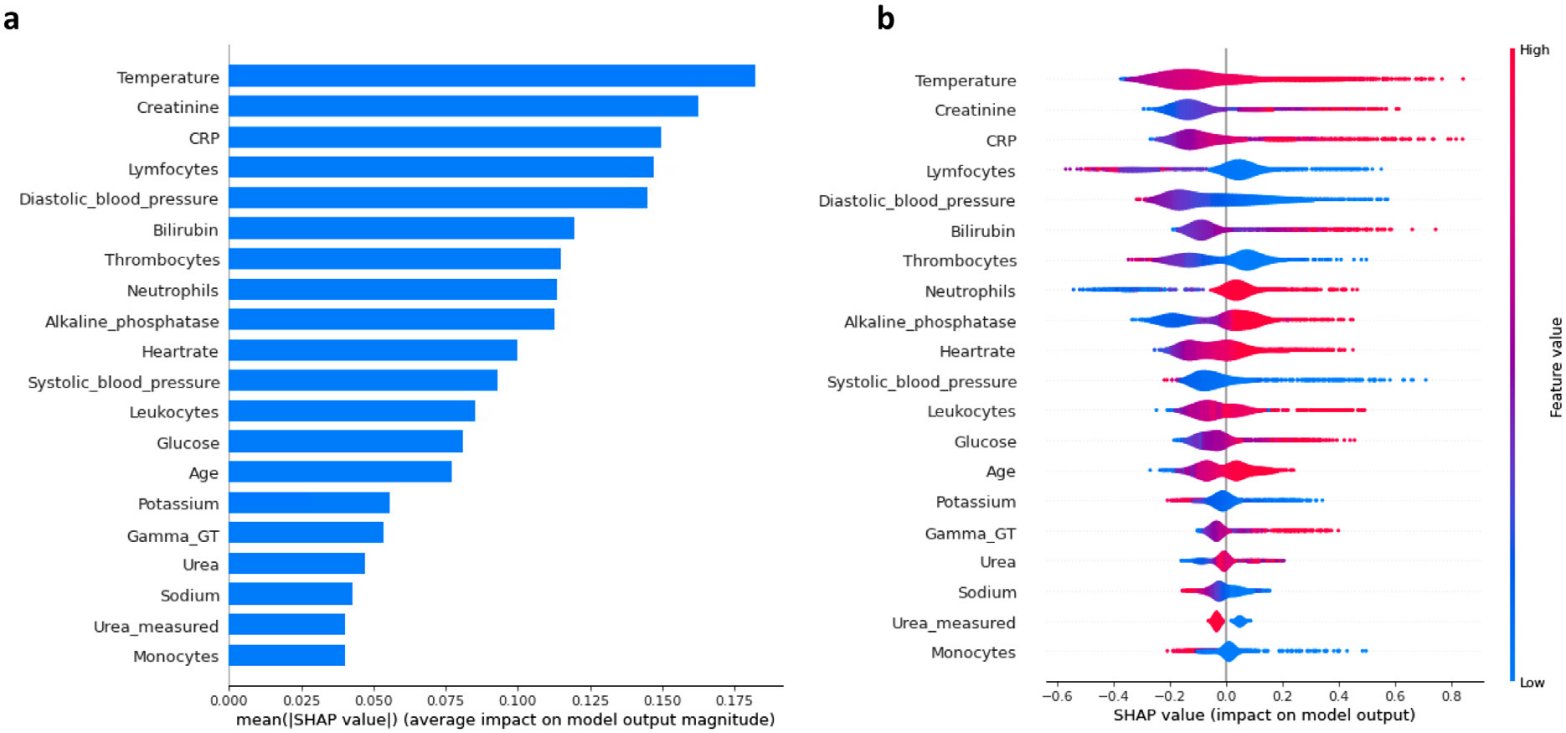
Feature importances of the top 20 predictors of the XGBoost model when predicting the outcome of blood cultures in the emergency department. According to the Shapley values, we see a. The average impact of the features on the prediction (either positive or negative). b. The local contributions of each feature for every prediction. Contributions on the left of 0 on the x-axis are associated with negative blood culture predictions, and contributions to the right of 0 on the x-axis are associated with positive blood culture predictions. The color represents the actual value of the feature at that particular prediction: blue represents a low actual value and red a high actual value.(For interpretation of the references to color in this figure legend, the reader is referred to the web version of this article.)

Figure 2a shows the twenty most important features in the XGBoost model in descending order. These features were a mixture of vital signs and laboratory results, while there was just one indicator feature among the top 20 (measurement of urea). Temperature, creatinine, and C-reactive protein were the top predictors. Figure 2b shows that low (blue) temperatures are generally associated with a negative blood culture (to the left of 0 on the x-axis), whereas high (red) temperatures are usually associated with positive blood cultures (to the right side of 0 on the x-axis).

### External validation of the prediction model

**Figure 3:**
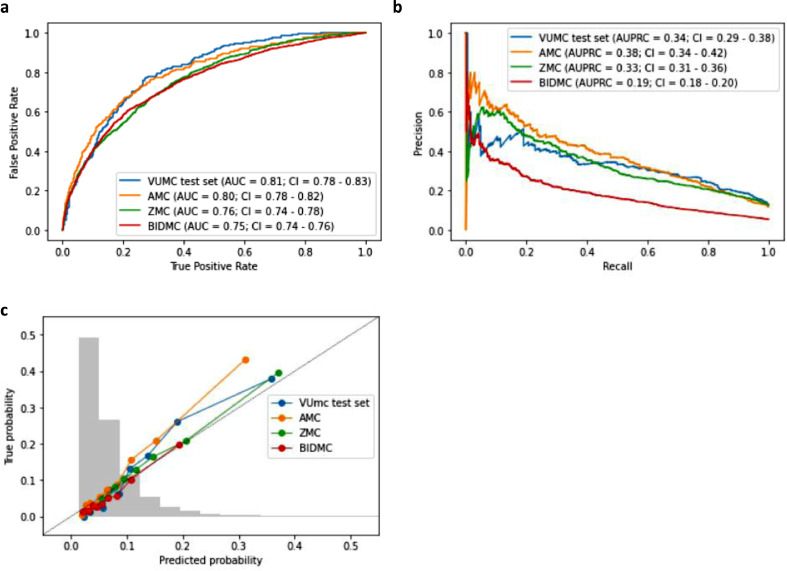
Discriminatory performance and calibration of the XGBoost model for predicting the outcome of blood cultures in the emergency department during validation in the VU Medical Center (VUMC) test set, Academic Medical Center (AMC), Zaans Medical Center (ZMC), and the Beth Israel Deaconess Medical Center (BIDMC). a. the area under the receiver operating characteristics curve (AUROC). b. the area under the precision-recall curve (AUPRC). c. the calibration plot of predicted probabilities compared with actual probabilities. In grey, we further see a histogram of the distribution of the various predictions of all four datasets combined.

We validated the performance on the VUMC test set and external datasets from a Dutch academic medical center (AMC), a Dutch regional teaching hospital (ZMC), and a large United States-based teaching hospital (BIDMC). Figure 3a shows that the model achieves an AUROC of 0.81 (95%-CI = 0.78–0.83) within the VUMC test set and retains AUROCs of 0.80 (95%-CI = 0.78–0.82), 0.76 (95%-CI = 0.74–0.78), and 0.75 (95%-CI = 0.74–0.76) in the AMC, ZMC, and BIDMC cohorts, respectively. Figure 3b shows that the AUPRC is 0.34 (95%-CI = 0.29–0.38) in the internal test set. The AUPRC is comparable in the AMC (0.38; 95%-CI = 0.34–0.42) and ZMC (0.33; 95%-CI = 0.31–0.36), but lower in the BIDMC (0.19; 95%-CI = 0.18–0.20). Overall, the model seems to be well-calibrated in all cohorts, as seen in Figure 3c.

### Prospective evaluation

**Figure 4:**
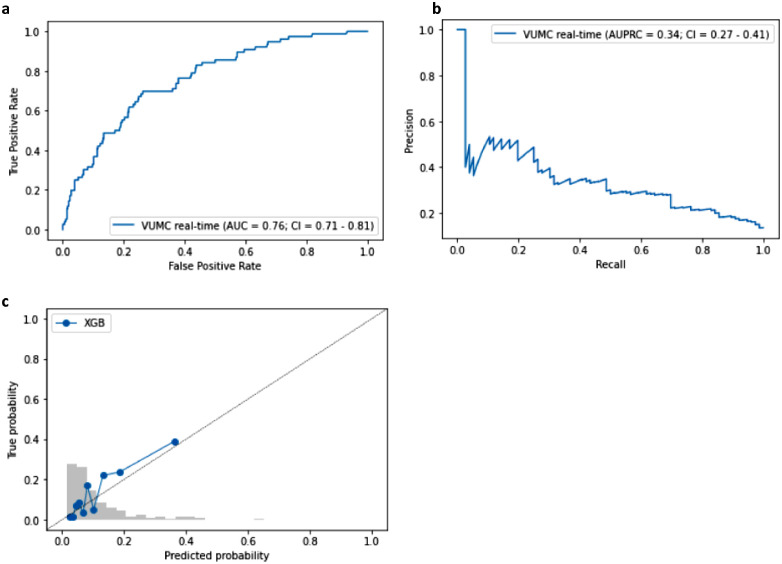
Prospective evaluation of the XGBoost prediction model for blood culture outcomes in the emergency department in the VU Medical Center. a. the area under the receiver operating characteristics curve (AUROC). b. the area under the precision-recall curve (AUPRC). c. the calibration plot of predicted probabilities compared with actual probabilities. In grey, we further see a histogram of the distribution of the predictions in the prospective evaluation.

Following the external validation, we integrated the XGBoost model into the EHR environment of the VUMC for a single-center real-time prospective evaluation. The model reached an AUROC of 0.76 (95%-CI = 0.71–0.81) and an AUPRC of 0.34 (95%-CI = 0.27–0.41) during the evaluation, as shown in Figure 4. In e-Figure 8, we display the pathogens found in the prospective evaluation cohort. If we had avoided blood culture draws or cancelled the analysis thereof in all patients with a risk of a positive culture of less than 5%, we would have avoided 179 (30.3%) blood cultures, of which 18 gave false-positive results, and missed 5 out of 76 pathogens in the cohort.

### Decision curve analysis

**Figure 5:**
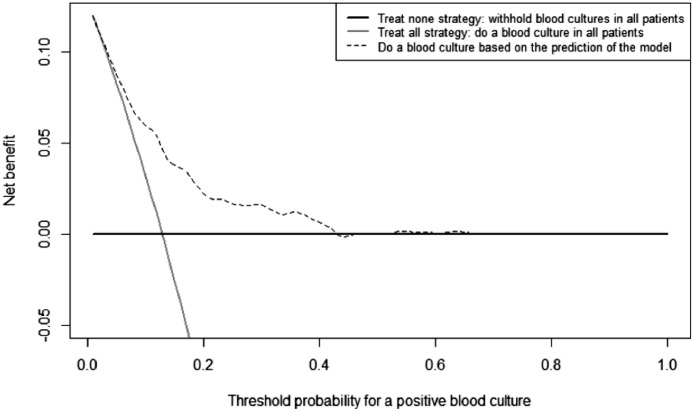
A net benefit decision curve analysis of the use of the XGBoost model to decrease blood culture testing during a prospective evaluation in the VUMC. Using the model provides a net benefit over a treat-all or treat none approach over an extensive range of potential cut-offs for converting the probability into an advice to do or withhold a blood culture.

The net benefit decision curve in Figure 5 shows that using the model to guide blood culture analyses in the ED could yield a net benefit over the current “culture all” approach across a range of threshold probabilities between 0.01 and 0.4 (40% probability of a positive culture) during the prospective real-time evaluation. According to Figure 5, the most significant benefits would be gained when thresholds between 0.1 and 0.2 would be used as cut-offs to withhold blood culture analyses. Although the net benefit at a threshold of 0.05 (5% probability of a positive culture) is much smaller, we presented the results of the prospective analysis at this cut-off since higher probabilities of missing a positive culture may not be accepted in practice. For more details on the decision curve analysis and net benefit calculations, see the e-Methods.

## Discussion

We created a machine learning prediction model for blood culture outcomes in the ED that performed well during internal and external validations. The XGBoost model reached an AUROC of 0.81 (95%-CI = 0.78–0.83) in the test set and up to 0.80 (95%-CI = 0.78–0.82) in external validations. Furthermore, a prospective real-time evaluation in the EHR environment of the VUMC showed that the model could retain a real-time performance with an AUROC of 0.76 (95%-CI = 0.71–0.81). A decision curve analysis showed that using the model in practice could provide a net benefit over the current approach across a large range of threshold probabilities for a positive blood culture.

Researchers have created several prediction models for blood culture outcomes in the past. Eliakim-Raz and colleagues presented fifteen such models in a 2015 systematic review.^25^ Of those models, only the Shapiro decision rule seems to have been implemented in practice.^26^ This striking gap between the development and implementation of prediction models has been apparent throughout the medical literature.^27^^,^^28^ A review by Fleuren et al. on machine learning readiness showed that 93% of machine learning papers discuss the development of a predictive model, while just 5% externally validate the models, and only 1% do real-time testing.^28^ In our study, we present a machine learning model that outperforms the current standard set by the Shapiro decision rule, and we complete stages one (problem identification) through six (real-time testing) of the machine learning readiness process.^28^ Further steps will be to acquaint physicians with the prediction model in a pilot study and then perform a randomized clinical trial to establish the model's effects in practice.

Various aspects of our analyses support the validity of the predictions. Firstly, the model retained its predictive performance during external validations in different hospitals, geographical locations, and periods. Data for validation represented a mix of academic and teaching hospitals in the Netherlands and the United States. The data were captured between 2011 and 2021, including a validation set (AMC) exclusively captured during the COVID-19 pandemic. The model performance decreased slightly when we used the model in the Dutch ZMC teaching hospital or the population of Boston's BIDMC. Given the substantial differences in patient populations, outcomes, and clinical protocols, this limited performance drop is reasonable. It suggests that minor recalibrations should suffice to obtain similar performances when using the model in different hospitals.^29^ The potential value of the predictions is strengthened by the comparable results we observed during the real-time prospective evaluation and is reinforced by feasible associations between the features in our model and the outcome. High temperatures, high C-reactive protein levels, and high neutrophil counts are associated with positive blood culture outcomes in our model. These variables are all associated with BSIs and infections in general in the literature.^2^^,^^7^^,^^26^ Levels of serum creatinine, bilirubin, and thrombocytes are also associated with the blood culture outcomes in our model, just as dysregulated vital sign measurements. In this case, the associations may represent critically ill patients with potential sepsis, in whom the prevalence of BSIs is known to be higher.^30^

Our machine learning tool can help reduce unnecessary blood culture analyses in the ED by identifying patients at low risk of a BSI, in whom we can safely withhold blood culture draws or cancel the analysis when the blood culture has already been sent to the lab. The consequent decrease in false-positive blood cultures may lead to lower resource use, shorter hospital stays, more appropriate use of antibiotics, and perhaps even lower in-hospital mortality.^9^^,^^12^, ^13^, ^14^ Choosing which threshold probability for a positive culture is acceptable as a cut-off for doing or withholding a blood culture in practice depends on the physicians’ preferences and concerns about the patient. The decision curve analysis showed that our model could provide net benefits across an extensive range of cut-offs. When using a threshold of just 5% for withholding a blood culture analysis, the model could already prevent over 30% of blood cultures, while missing a true-positive culture in 1% of cases. A clinical trial and health economic assessment are needed to fully capture the associated health- and cost gains. Choosing a higher threshold as a cut-off would help avoid even more unnecessary blood cultures, but at the cost of missing additional true positives. In the worst-case scenario, withholding blood culture sampling could lead to a missed opportunity to identify a pathogen. We showed that this scenario rarely occurs at the 5% probability threshold during the prospective evaluation. And even when it did, it could still be that the pathogens were also found through other cultures (e.g., the missed *E. coli* may also have been found through urine cultures), or that the treatment strategy would have been the same regardless of the finding of the BSI. To better understand the workflow alterations that come with using our model to avoid blood culture analyses in low-risk patients, we present two cases in Textbox 1. Strictly, all available blood culture prediction tools, including the Shapiro rule, can only validly be used in situations where the physician has already decided to do a blood culture, as they are derived from datasets of patients who underwent a blood culture draw. A valid prediction will thus need to override a clinical decision that the physician already made.

A primary limitation of this study is that we were unable to reliably examine the performance of our tool in subgroups of the population with specific comorbidities or medications. We would need data stored in free-text fields for this analysis. Arguably, the performance of our model could be worse for immunocompromised patients. This limitation warrants a detailed investigation when we test the model in a clinical trial, where we could reliably capture this information. Furthermore, we defined certain microorganisms as contaminants, while they may still represent a pathogen in specific patient groups. Examples are clinically relevant infections with coagulase-negative staphylococci (CoNS) in central line-associated BSI and prosthetic cardiac valve infections. The model must be validated separately for these patient groups in a clinical trial. A final limitation of our study is that the performance of static prediction models, including our model, could vary over time due to changes in the patient characteristics or the prevalence of positive blood cultures. When we introduce the model in practice, we expect a change in the blood culture positivity rate, as physicians may be tempted to use the model in an even broader population of patients. The performance should thus be closely monitored during implementation. We hope that future developments will make it possible to more easily implement dynamic models that can be updated in real-time and adjust predictions based on new outcome prevalence and cohort characteristics.

In conclusion, we developed a machine learning model to predict blood culture outcomes in the ED, which retained its performance during external validation and real-time prospective evaluation. Our model can identify patients at low risk of having a positive blood culture. Using the model in practice could reduce the number of unnecessary blood cultures by at least 30% and thus avoid the hidden costs of false-positive culture results.

## Contributors

MS, AWB, and PWBN conceptualized this study. MS, AWB, FCB, ML, and RPS curated the data. MS, AWB, FCB, TCM, FH, RPS, RJ, WJW, and PWBN collectively investigated the data and decided on the methodology to be used. MS, AWB, FCB, and HPS conducted the formal analyses. MS and ML developed the resources. FCB, TCM, FH, RPS, RJ, WJW, and PWBN supervised various parts of the research process within their expertise. MS, AWB, TCM, FH, RJ, WJW, and PWBN acquired funding. MS, AWB, TCM, and HPS drafted the original manuscript. MS, AWB, FCB, TCM, ML, HPS, FH, RPS, DJ, WJW, and PWBN reviewed, edited, and agreed with the final version of the manuscript.

## Data sharing statement

Participant data underlying the results of this study can be shared after de-identification. The data can be requested following publication of this work. The data can be shared with researchers who provide a methodologically sound proposal, which is allowed under our local privacy regulations. Proposals should be directed to the corresponding author and requestors will need to sign a data access agreement. Part of the data is available to all researchers through the MIMIC-IV-ED database (https://physionet.org/content/mimic-iv-ed/1.0)

## Declaration of interests

The authors declare no competing interests pertaining to the submitted work.

## Appendix Supplementary materials

Image, application 1

Image, application 2

Image, application 3
