# Supplementary material for: Diagnostic stewardship for blood cultures in the emergency department: A multicenter validation and prospective evaluation of a machine learning prediction tool
Source: eBioMedicine. 2022 Jul 16;82:104176. doi: 10.1016/j.ebiom.2022.104176 (PMC9294655; doi:10.1016/j.ebiom.2022.104176)
Supplement: Supplementary file 1 [file mmc1.docx]

Supplementary appendix

Contents

[e-Methods 2](#_Toc106027701)

[A. Patient selection 2](#_Toc106027702)

[B. Variable selection 2](#_Toc106027703)

[C. Variable cleaning 2](#_Toc106027704)

[D. Approach to missing data 2](#_Toc106027705)

[E. Outcome processing and definitions 3](#_Toc106027706)

[F. Model training 3](#_Toc106027707)

[G. Logistic regression model outcomes 3](#_Toc106027708)

[H. Decision curve analysis 3](#_Toc106027709)

[e-Figures 4](#_Toc106027710)

[e-figure 1: Overview of the number of emergency department visits at each stage of the data cleaning procedure in the different cohorts. 4](#_Toc106027711)

[e-Figure 2. The performance of the logistic regression model during training. 5](#_Toc106027712)

[e-Figure 3. The performance of the logistic regression model during validation. 6](#_Toc106027713)

[e-Figure 4. The performance of the XGBoost model in a sensitivity analysis using iterative imputation. 7](#_Toc106027714)

[e-Figure 5. The performance of the XGBoost model in a sensitivity analysis defining contamination based on the number of positive bottles. 8](#_Toc106027715)

[e-Figure 6. The coefficients of the logistic regression model for all predictor variables. 9](#_Toc106027716)

[e-Figure 7. An overview of the top 10 microorganisms found in the various cohorts. 10](#_Toc106027717)

[e-Figure 8. An overview of the microorganisms found during the prospective evaluation. 11](#_Toc106027718)

[e-Tables 12](#_Toc106027719)

[e-Table 1. The logical minimum and maximum per variable. 12](#_Toc106027720)

[e-Table 2. The grid of the search for optimal hyperparameters during model training. 13](#_Toc106027721)

[e-Table 3. An overview of the percentage of imputed values in the various cohorts. 14](#_Toc106027722)

[e-Table 4. Summary characteristics of the features stratified by blood culture outcome 15](#_Toc106027723)

[e-Table 5. Microorganisms categorized as contaminants. 16](#_Toc106027724)

[References 17](#_Toc106027725)

# e-Methods

### A. Patient selection

For the VUMC, AMC, and BIDMC cohorts, we received anonymized extracts of the complete emergency department (ED) population to further filter based on our specific study. We processed all cohorts similarly and selected all adult patients for whom a blood culture was registered in the Electronic Health Records (EHR) system during the ED stay, based on the associated time stamps. The ZMC dataset was created by local business intelligence specialists and only included patients who had a blood culture taken during the ED stay. Therefore, we did not need to select these further. We excluded patients with ED stays of over 24 hours, as these may have been caused by errors in the EHR registration times or would be rare situations.

After the feature selection procedure (described below), we selected only the patients for whom at least 20% of the vital signs and 20% of the laboratory results were available, ensuring a minimum of four actual laboratory results and two vital signs as the basis for the prediction. These selections would also be made in the prospective evaluation and filters out irregularities in the data as it confirmed that a nurse had actively seen the patient and recorded vital sign data (with just one vital sign, this could have been a single automated heart rate measurement by the monitor) and also confirmed that the physician had actively ordered diagnostic tests other than the blood culture. e-Figure 1 shows an overview of the number of ED visits at each stage of the selection process.

### B. Variable selection

The variable selection was carried out in the VUMC cohort, as this was the development set. We selected age, sex, laboratory results, and vital sign measurements, as these variable groups were the primary drivers of the predictions in the initial model [1]. For the laboratory results and vital sign measurements, we selected only those whose outcomes were registered in the EHR system before the end of an ED visit. We averaged the results if there were multiple measurements of a variable during one visit. From the complete set of laboratory results measured in the VUMC population of patients who underwent a blood culture draw in the ED, we only used variables measured in over 50% of the population. We further disregarded the estimated Glomerular Filtration Rate (eGFR) as a predictor variable, as it could be calculated differently in different hospitals. When variables were measured in 30% to 50% of the population, we created indicator variables to denote when these were measured without using the actual values. This was the case for albumin, aspartate aminotransferase, and lactate dehydrogenase.

### C. Variable cleaning

As we work with EHR data, there can be erroneous measurements included in the data. Before finalizing the dataset, we excluded values that were deemed physiologically implausible. Cut-offs for these implausible values were based on the cut-offs by the VUMC clinical chemistry department, earlier work on this topic, or based on expert opinion [1]. The logical minimum and maximum per feature are presented in e-Table 1. For values in the BIDMC cohort, from the online MIMIC-IV-ED database, we had to convert some of the measurements to the SI units used in the Dutch populations.

### D. Approach to missing data

After finalizing the list of predictor variables, we added indicator variables for all laboratory tests and vital signs to indicate whether they were measured or missing on a patient level as we would need to impute the missing data during the modeling phase. We chose to impute missing values with the median of the training set, as median imputation combined with indicator variables is a practical approach to handle missing values and is especially effective with data missing not at random, as is the case in our data [2]. The percentage of missing values per variable per cohort is presented in e-Table 3.

As a sensitivity analysis, we also ran the XGBoost training on a dataset imputed using an iterative imputer [3]. The iterative imputer implements Multivariate Imputation by Chained Equations (MICE) but only returns a single imputation [4]. The results of this analysis are presented in e-Figure 4, which shows nearly identical results to the median imputation strategy. Since there was no difference in these outcomes, we opted to use the more straightforward and easier to explain median imputation approach.

### E. Outcome processing and definitions

The outcomes of the blood cultures could be textually different between the various cohorts. To aid reproducibility and robustness, we used the AMR package in the R statistical software to reclassify the blood culture outcomes of the various cohorts to standardized family names and microbe names [5]. We defined likely contaminants based on previous literature and classified those as negative cultures [6–9]. The list of microorganisms categorized as likely contaminants can be seen in e-Table 4, and e-Figure 7 visualizes the top 10 organisms found in the different cohorts.

Since classification based on the microorganism but not the clinical context could introduce a bias, we experimented with a different approach. We defined contamination based on the number of bottles with the likely contaminants as a sensitivity analysis. When likely contaminants were found in over 50% of culture bottles, they were classified as positive in this sensitivity analysis. One problem with this approach was that 57.8% of the population had only one set (of two bottles) of blood cultures taken during their visit, compared with 34.9% who had two sets of blood cultures drawn, and just 7.3% with three or more sets. In the 57.8% of cases with just one set, and thus two bottles, a likely contaminant was already classified as positive if they were present in one bottle. Therefore, the total number of cultures classified as positive increased substantially from 922 to 1121. e-Figure 5 shows that the model’s performance with these outcome labels was considerably worse in the VUMC training cohort, with an AUROC of 0.75. Since this approach would also present difficulties when only an overall blood culture outcome would be shown in the EHR system, instead of per bottle, it was deemed inferior.

### F. Model training

The VUMC cohort was split into a training (80%) and test (20%) set, stratified by outcomes. After scaling and imputing the data through a pipeline, we trained a logistic regression and XGBoost model on the VUMC training cohort [10–14]. The optimal hyperparameters were found through a fivefold cross-validated grid search, of which further details are presented in e-Table 1 [15, 16].

### G. Logistic regression model outcomes

Since the XGBoost model consistently outperformed the logistic regression, we presented only the XGBoost model performance in the main paper with a prospective evaluation. Here we present the logistic regression model results. During the training phase, the logistic regression model reached an Area Under the Receiver Operating Characteristics curve (AUROC) of 0.77 and an Area Under the Precision-Recall Curve of 0.31, as visualized in e-Figure 1A and 1B. e-Figure 1C shows that the model was well-calibrated. The same results for the validation phase are shown in e-Figure 2, with AUROCs ranging between 0.73-0.79 and AUPRCs between 0.16-0.32. In Figure 3, we see the importance of all the features in the logistic regression model based on the coefficients.

### H. Decision curve analysis

The benefit of using a prediction model in practice depends on the balance between the clinical benefit of doing a blood culture (i.e., not missing a positive culture, being able to narrow the antibiotic spectrum) and the adverse side effects (i.e., more contaminated cultures with associated resource use, antibiotics use, increased length of hospital stay) [17, 18]. As our model outputs a probability of a positive blood culture, we can define a threshold probability where the expected benefit of taking the blood culture equals the expected benefit of withholding the culture. However, this also depends on the likelihood of the outcomes and the judgment of these outcomes by the physician. The net benefit can be calculated as (TP― w*FP)/N, where TP is the number of true-positive decisions, FP is the number of false-positive decisions, N is the total number of patients and w is a weight equal to the odds of the cut-off given by the threshold probability [17, 18]. The numbers outputted from this net benefit calculation provide a purely theoretical evaluation that does not translate perfectly into the clinical setting. However, it gives a robust evaluation of the potential approaches. At a threshold probability for a positive culture of 5%, the model achieves a net benefit of 0.088 over withholding cultures for all patients, equivalent to detecting 8.8 true-positive blood cultures per 100 patients without increasing false positives. Furthermore, the net benefit of using the model at a probability threshold of 5% is 0.006 higher than with doing blood cultures in all patients (0.088 - 0.082 = 0.006). Using the model with this cut-off would thus result in finding six additional true-positive blood culture per 1000 patients without increasing the number of false positives, compared with the current “culture all” approach. Notably, the “culture all approach” line represents the relation between the threshold used to intervene (shown on the x-axis), and the prevalence of positive blood cultures in the studied population.

# e-Figures

### e-figure 1: Overview of the number of emergency department visits at each stage of the data cleaning procedure in the different cohorts.

ED = Emergency Department; BC = Blood culture; VUMC = VU Medical Center; AMC = Academic Medical Center; ZMC = Zaans Medical Center; BIDMC = Beth Israel Deaconess Medical Center. Sufficient lab/vitals indicates that at least 20% of the vital sign measurements and 20% of the laboratory results were registered in the system before the end of an ED visit. The percentage of visits with sufficient data is considerable less in the BIDMC cohort, which may reflect differences in diagnostic protocols in the ED.

### e-Figure 2. The performance of the logistic regression model during training.

During training in the VUMC cohort, we see A. The Area Under the curve of the Receiver Operating Characteristics (AUROC). B. The Area Under the Precision-Recall Curve (AUPRC). C. The model calibration with in grey the distribution of the predictions.

**A B**


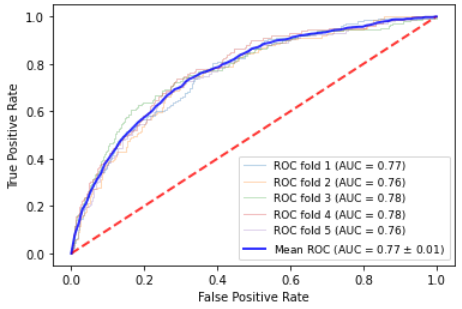
  **
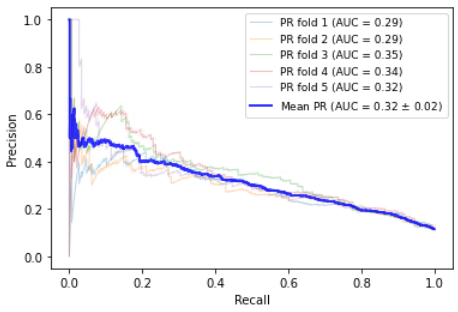
**

**C**

**
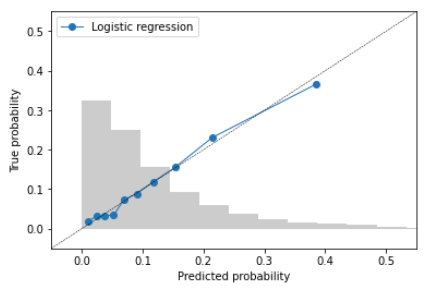
**

### e-Figure 3. The performance of the logistic regression model during validation.

During validation in the VUMC test set, AMC, ZMC, and BIDMC, we see A. The Area Under the curve of the Receiver Operating Characteristics (AUROC). B. The Area Under the Precision-Recall Curve (AUPRC). C. The model calibration with the distribution of the predictions in all cohorts combined in grey.

**A B**

**
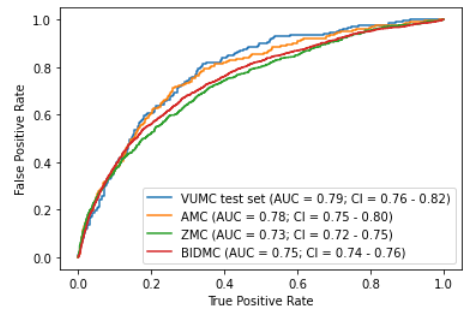

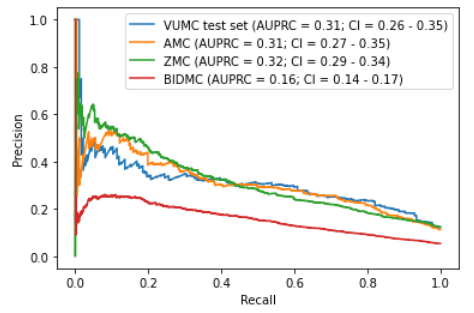
**

**C**

**
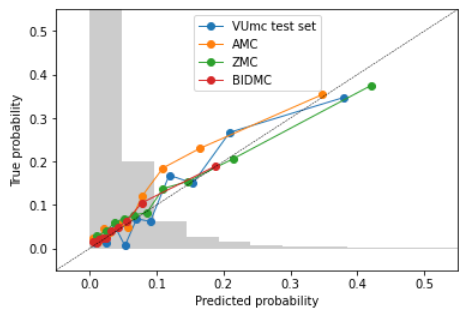
**

### e-Figure 4. The performance of the XGBoost model in a sensitivity analysis using iterative imputation.

Discriminatory performance and calibration of the XGBoost model for predicting the outcome of blood cultures in the emergency department. Instead of using median imputation, this analysis uses an iterative imputer. During the training phase in the VUMC training set, we see A. the Area Under the Receiver Operating Characteristics curve (AUROC). B. the Area Under the Precision-Recall Curve (AUPRC). C. the calibration plot of predicted probabilities compared with actual probabilities. In grey, we further see a histogram of the distribution of the predictions in the training set in this figure.

**A B**


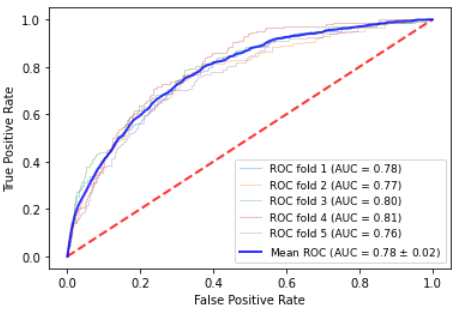

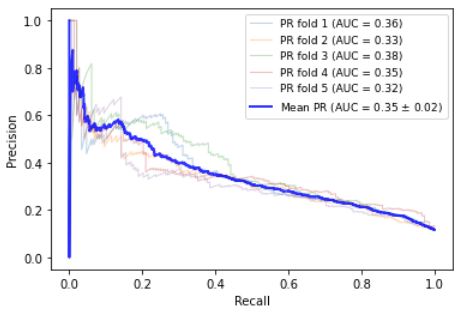


**C**


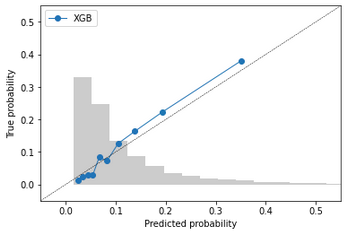


### e-Figure 5. The performance of the XGBoost model in a sensitivity analysis defining contamination based on the number of positive bottles.

Discriminatory performance and calibration of the XGBoost model for predicting the outcome of blood cultures in the emergency department. Instead of using a standard definition of contamination based on the list in e-Table 4, this analysis also considers the number of positive bottles with this likely contaminant. During the training phase in the VUMC training set, we see A. the Area Under the Receiver Operating Characteristics curve (AUROC). B. the Area Under the Precision-Recall Curve (AUPRC). C. the calibration plot of predicted probabilities compared with actual probabilities. In grey, we further see a histogram of the distribution of the predictions in the training set in this figure.

**A B**


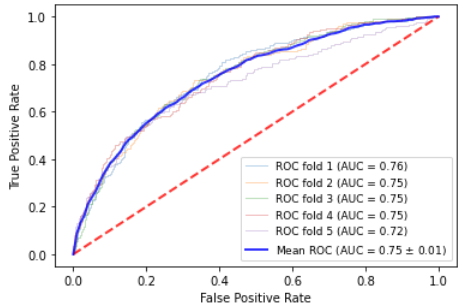

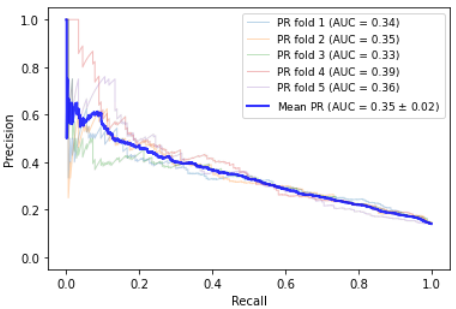


**C**


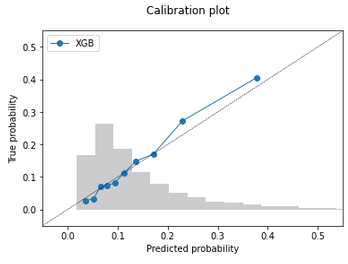


### e-Figure 6. The coefficients of the logistic regression model for all predictor variables.

This figure presents the coefficients of all the features in the logistic regression model. The features with a “_measured” suffix are the binary indicator variables (was this variable measured (1) or imputed (0). Features with a negative coefficient predict a lower chance of a positive blood culture with higher actual values of that specific variable.


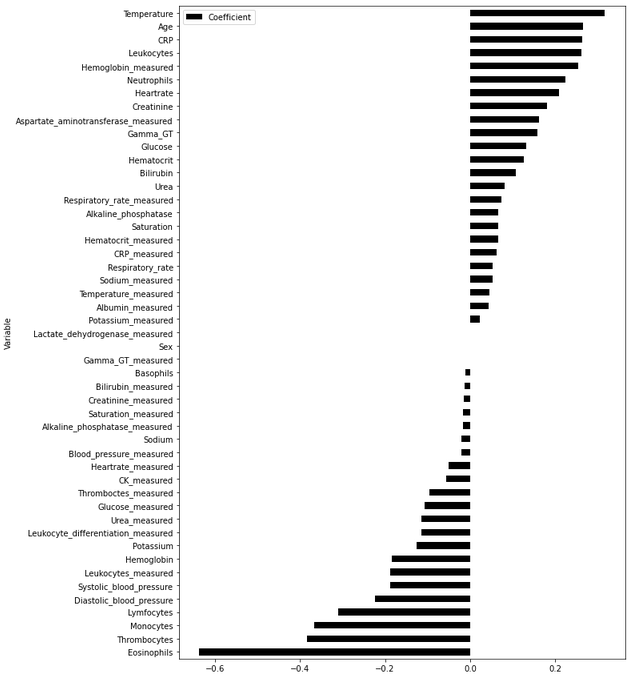


### e-Figure 7. An overview of the top 10 microorganisms found in the various cohorts.

This faceted figure presents the top 10 pathogenic microorganisms found in the various cohorts. We only present pathogens and not contaminants. The number of pathogens found is higher than the number of positive tests, as some cultures contain multiple microorganisms. GBS = Group B Streptococci.


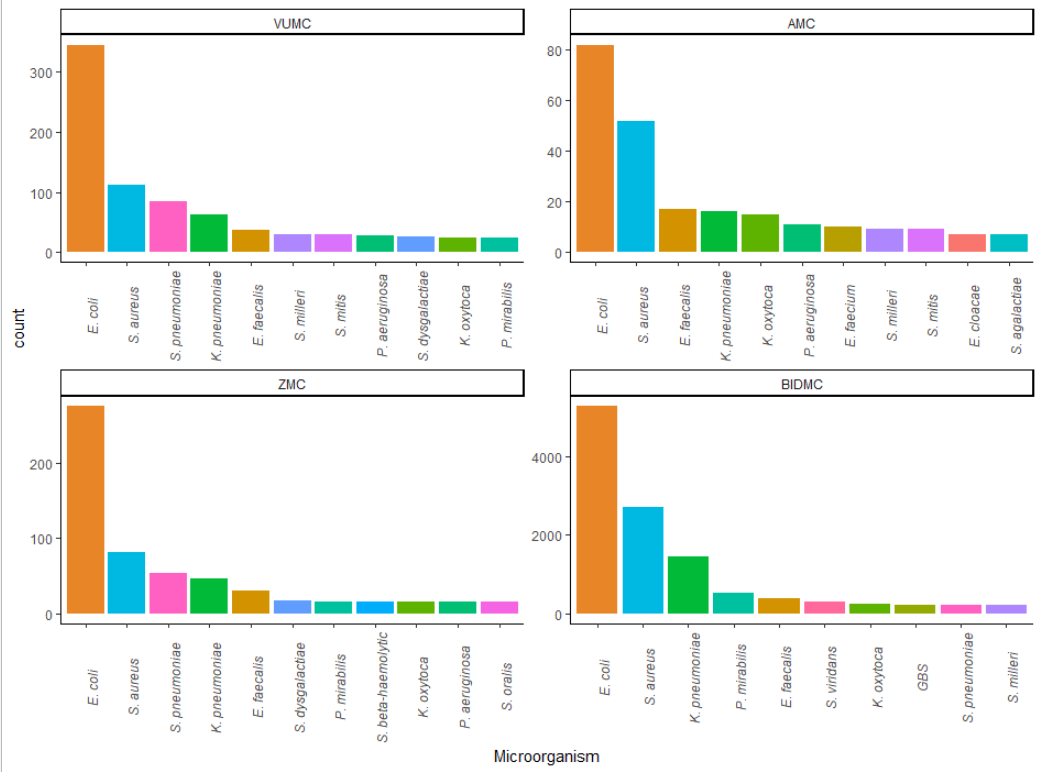


### e-Figure 8. An overview of the microorganisms found during the prospective evaluation.

The figure presents the pathogens found during the prospective evaluation and splits them based on the risk prediction by the model. The figure shows that only five pathogens were found in the low-risk group. In the high-risk group, 76 pathogens were found, of which the top 10 are presented here.


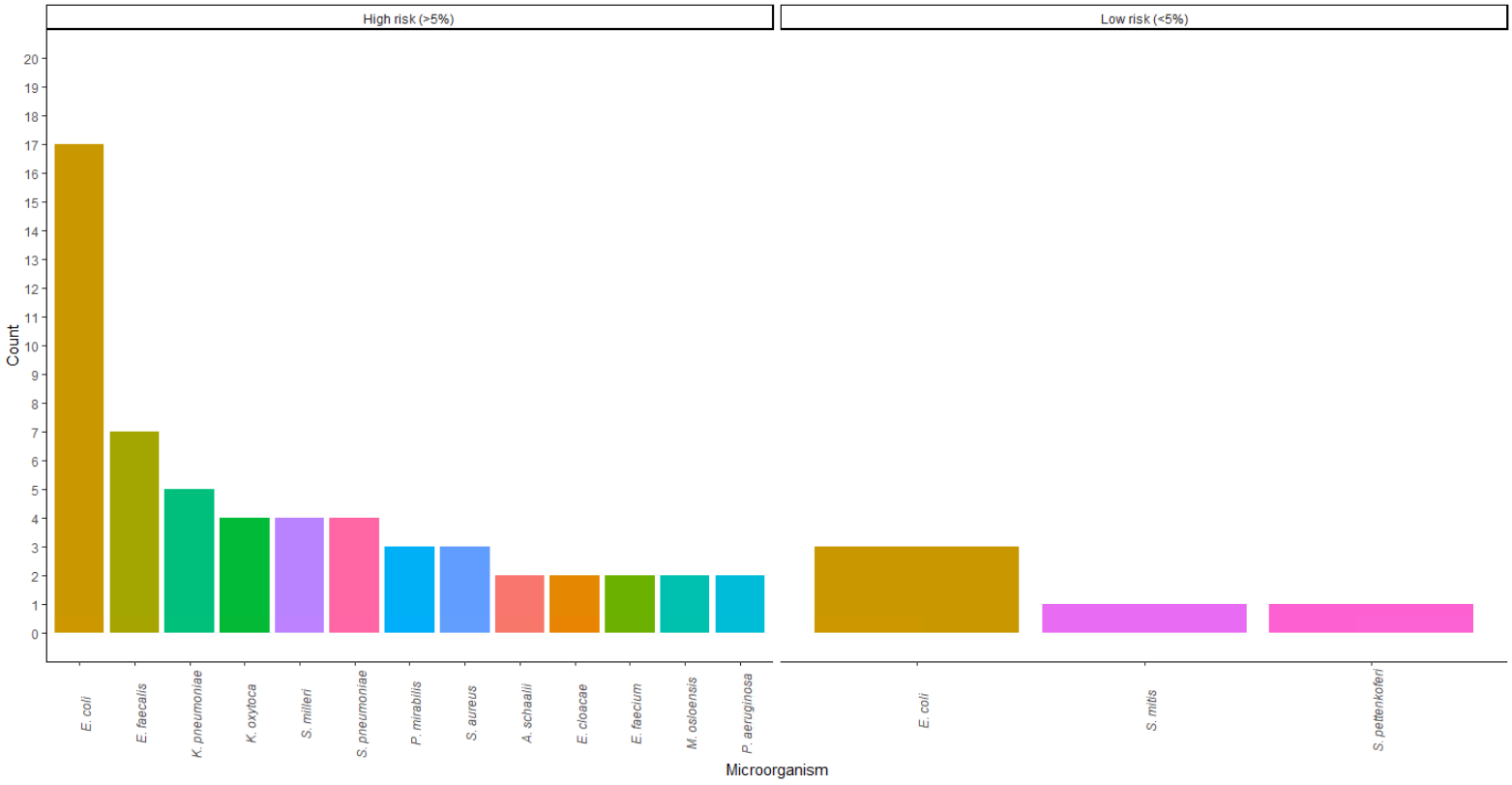


# e-Tables

### e-Table 1. The logical minimum and maximum per variable.

| **Parameter** | **Minimum** | **Maximum** |
| --- | --- | --- |
| Alkaline Phosphatase | 10 | 2500 |
| Basophils | 0.001 | 6 |
| Bilirubin | 2 | 1000 |
| Creatinine | 10 | 2500 |
| C-Reactive Protein | 0.01 | 700 |
| Eosinophils | 0 | 45 |
| Gamma GT | 3 | 6000 |
| Glucose | 1 | 120 |
| Hemoglobin | 1 | 17 |
| Hematocrit | 0.05 | 0.75 |
| Leukoctyes | 0.001 | 60 |
| Lymfocytes | 0.001 | 22 |
| Monocytes | 0.001 | 15 |
| Neutrophils | 0.001 | 60 |
| Potassium | 1 | 10 |
| Sodium | 95 | 180 |
| Thrombocytes | 0.1 | 2400 |
| Urea | 0.5 | 95 |
| Heartrate | 1 | 300 |
| Systolic blood pressure | 40 | 250 |
| Diastolic blood pressure | 40 | 250 |
| Temperature | 28 | 45 |
| Respiratory rate | 1 | 80 |
| Saturation | 15 | 100 |

### e-Table 2. The grid of the search for optimal hyperparameters during model training.

| **Hyperparameter** | **Model** | **Grid** | **Optimized value** |
| --- | --- | --- | --- |
| Learning rate | XGBoost | [0.01, 0.05, 0.1] | 0.01 |
| Gamma | XGBoost | [1, 1.5, 2] | 1 |
| Minimum child weight | XGBoost | [1, 5, 10] | 1 |
| Maximum depth | XGBoost | [3, 5, 7] | 7 |
| Subsample | XGBoost | [0.4, 0.6, 0.8, 1] | 0.4 |
| Colsample by tree | XGBoost | [0.6, 0.8, 1] | 0.6 |
| C | Logistic regression | [0.01, 0.05, 0.1, 0.5, 1] | 0.5 |
| Solver | Logistic regression | [lbfgs, newton-cg, liblineair, saga] | saga |
| Penalty | Logistic regression | [L1, L2] | L1 |

### e-Table 3. An overview of the percentage of imputed values in the various cohorts.

The percentage of imputed values per variable per cohort. The final row shows the average rate of imputed values in the complete cohort.


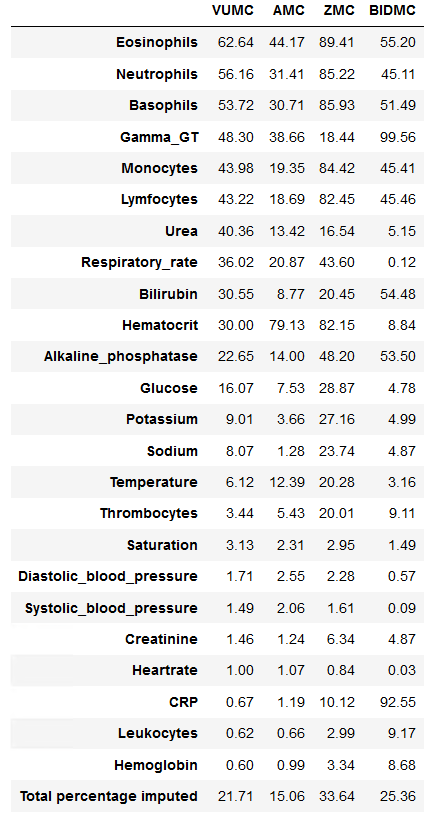


### e-Table 4. Summary characteristics of the features stratified by blood culture outcome

A summary of all the real values of the features in the training cohort (VUmc).

| **Characteristic** | **Culture negative (n=5683)** | **Culture positive (738)** |
| --- | --- | --- |
| Age (median, IQR) | 66 (52-76) | 69 (58-78) |
| Sex (%, female) | 43.2 | 40.4 |
| Alkaline phosphatase (median, IQR) | 87.5 (68.1-123.4) | 104.5 (78.8-177.9) |
| Basophils (median, IQR) | 0.03 (0.02-0.05) | 0.03 (0.02-0.05) |
| Bilirubin (median, IQR) | 8.7 (5.9-13.6) | 13.3 (7.9-22.2) |
| Creatinine (median, IQR) | 93.1 (64.8-114.6) | 103.1 (72.7-152.1) |
| C-Reactive Protein (median, IQR) | 59 (19-132) | 104 (40-213) |
| Eosinophils (median, IQR) | 0.06 (0.02-0.15) | 0.03 (0.01-0.08) |
| Gamma Glutamyltransferase (median, IQR) | 44 (24-106) | 71.5 (31.1-189.8) |
| Glucose (median, IQR) | 6.81 (5.87-8.53) | 7.5 (6.1-9.8) |
| Hemoglobin (median, IQR) | 7.7 (6.7-8.6) | 7.3 (6.4-8.4) |
| Hematocrit (median, IQR) | 0.38 (0.33-0.42) | 0.36 (0.31-0.41) |
| Leukocytes (median, IQR) | 10.2 (6.9-14.3) | 11.9 (8.0-16.6) |
| Lymphocytes (median, IQR) | 0.96 (0.57-1.50) | 0.55 (0.32-0.91) |
| Monocytes (median, IQR) | 0.71 (0.44-1.02) | 0.61 (0.29-0.99) |
| Neutrophils (median, IQR) | 7.2 (4.6-10.6) | 10.8 (7.4-14.2) |
| Potassium (median, IQR) | 4.1 (3.7-4.4) | 4.0 (3.7-4.4) |
| Sodium (median, IQR) | 137.4 (134.7-139.7) | 136.4 (133.6-139.0) |
| Thrombocytes (median, IQR) | 236 (176-315) | 209 (149-273) |
| Urea (median, IQR) | 6.4 (4.5-9.6) | 9.2 (5.9-13.9) |
| Heartrate (/min, median, IQR) | 93 (81-105) | 98.8 (86.8-111.5) |
| Systolic blood pressure (mmHg, median, IQR) | 125 (111-141) | 116 (102-133) |
| Diastolic blood pressure (mmHg, median, IQR) | 75 (66-84) | 69 (60-77) |
| Temperature (Celsius, median, IQR) | 37.7 (36.9-38.4) | 38.1 (37.3-38.9) |
| Respiratory rate (/min, median, IQR) | 20 (16-25) | 22 (18-26) |
| Saturation (%, median, IQR) | 96 (94.5-98) | 36 (94-97) |

### e-Table 5. Microorganisms categorized as contaminants.

A list of all microorganisms we categorized as contamination based on the literature [1, 6–9].

| **Contaminants** |
| --- |
| *Staphylococci* (other than *S. aureus/S. lugdunensis/S. saprophyticus/S. pettenkoferi*) |
| *Micrococcus spp.* |
| *Propionibacterium spp.* |
| *Corynebacterium spp.* |
| *Bacillus spp.* |
| *Clostridium perfringens* |

# References

1. Boerman AW, Schinkel M, Meijerink L, et al (2022) Using machine learning to predict blood culture outcomes in the emergency department: a single-centre, retrospective, observational study. BMJ Open 12:53332

2. Choi J, Dekkers OM, le Cessie S (2019) A comparison of different methods to handle missing data in the context of propensity score analysis. Eur J Epidemiol 34:23–36

3. 6.4. Imputation of missing values — scikit-learn 1.0.1 documentation. https://scikit-learn.org/stable/modules/impute.html. Accessed 15 Dec 2021

4. sklearn.impute.IterativeImputer — scikit-learn 1.0.1 documentation. https://scikit-learn.org/stable/modules/generated/sklearn.impute.IterativeImputer.html. Accessed 15 Dec 2021

5. Parte AC, Carbasse JS, Meier-Kolthoff JP, Reimer LC, Göker M (2021) Antimicrobial Resistance Data Analysis [R package AMR version 1.7.1]. Int J Syst Evol Microbiol 70:5607–5612

6. Coburn B, Morris AM, Tomlinson G, Detsky AS (2012) Does this adult patient with suspected bacteremia require blood cultures? JAMA - J Am Med Assoc 308:502–511

7. Nannan Panday RS, Wang S, Van De Ven PM, Hekker TAM, Alam N, Nanayakkara PWB (2019) Evaluation of blood culture epidemiology and efficiency in a large European teaching hospital. PLoS One. https://doi.org/10.1371/journal.pone.0214052

8. Dargère S, Cormier H, Verdon R (2018) Contaminants in blood cultures: importance, implications, interpretation and prevention. Clin Microbiol Infect 24:964–969

9. Harvey DJ, Albert S (2013) Standardized definition of contamination and evidence-based target necessary for high-quality blood culture contamination rate audit. J Hosp Infect 83:265–266

10. sklearn.linear_model.LogisticRegression — scikit-learn 1.0 documentation. https://scikit-learn.org/stable/modules/generated/sklearn.linear_model.LogisticRegression.html. Accessed 22 Oct 2021

11. Python Package Introduction — xgboost 1.6.0-dev documentation. https://xgboost.readthedocs.io/en/latest/python/python_intro.html. Accessed 22 Oct 2021

12. sklearn.preprocessing.StandardScaler — scikit-learn 1.0 documentation. https://scikit-learn.org/stable/modules/generated/sklearn.preprocessing.StandardScaler.html. Accessed 22 Oct 2021

13. sklearn.impute.SimpleImputer — scikit-learn 1.0 documentation. https://scikit-learn.org/stable/modules/generated/sklearn.impute.SimpleImputer.html. Accessed 22 Oct 2021

14. sklearn.pipeline.Pipeline — scikit-learn 1.0 documentation. https://scikit-learn.org/stable/modules/generated/sklearn.pipeline.Pipeline.html. Accessed 22 Oct 2021

15. sklearn.model_selection.StratifiedKFold — scikit-learn 1.0 documentation. https://scikit-learn.org/stable/modules/generated/sklearn.model_selection.StratifiedKFold.html?highlight=stratified kfold#sklearn.model_selection.StratifiedKFold. Accessed 22 Oct 2021

16. sklearn.model_selection.GridSearchCV — scikit-learn 1.0 documentation. https://scikit-learn.org/stable/modules/generated/sklearn.model_selection.GridSearchCV.html. Accessed 22 Oct 2021

17. Vickers AJ, Elkin EB (2016) Decision Curve Analysis: A Novel Method for Evaluating Prediction Models: http://dx.doi.org.vu-nl.idm.oclc.org/101177/0272989X06295361 26:565–574

18. Vickers AJ, van Calster B, Steyerberg EW (2019) A simple, step-by-step guide to interpreting decision curve analysis. Diagnostic Progn Res 2019 31 3:1–8
