# Supplementary material for: Diagnostic stewardship for blood cultures in the emergency department: A multicenter validation and prospective evaluation of a machine learning prediction tool
Source: eBioMedicine. 2022 Jul 16;82:104176. doi: 10.1016/j.ebiom.2022.104176 (PMC9294655; doi:10.1016/j.ebiom.2022.104176)
Supplement: Supplementary file 3 [file mmc3.docx]

Supplementary appendix_clean: Supplementary appendix

TRIPOD checklist: TRIPOD Checklist

Textbox = Textbox 1
